# Power in the Pulpit

**Published:** 1858-02

**Authors:** 


### "POWER IN THE PULPIT"

Is certainly a very suggestive expression in the *Presbyterian Expositor*, of Chicago, under the head of *Ministerial Calls.* Its able editor, N. L. Rice, D.D., contends that there is a great lack of that power in these degenerate times, and that this deficiency is a sufficient cause for the fact that multitudes of congregations all over the country, of all denominations, are "vacant," have no settled minister, and, as a legitimate consequence, begin to decline in efficiency, dwindle in numbers, and go out in the night of disruption. In the Atlantic States, of the North especially, is this decline observable, to the sorrow of all good men. The cause is three-fold: Physiological, Pecuniary, and Ecclesiastical.

*Physiological*, in that the clergy, as a class, are not men of vigorous bodily health.

*Pecuniary*, in that they are not paid with sufficient liberality to enable them to devote their undivided energies to the duties of their calling.

*Ecclesiastically*, in that proper attention is not paid to the early indoctrination of the children of the Church, into the distinctive sentiments of that particular branch of the Church of which they make a part.

Physical power, mental power, moral power—these three are essential to a successful ministry, and upon these should every young man steadily fix his eye, from the very first moment of his entering the "Seminary."

To have "power in the pulpit," we must have men of vigorous health, which will enable them to perform that large amount of physical duty which devolves on a clergyman who properly visits the sick, the poor, the fainting, the desponding of his charge, and attends to the great variety of "meetings," at which his presence and countenance are considered indispensable. Rain or snow, hail-storm or hurricane, at midnight or the cock crowing, he must instantly repair to the chamber of the dying, whether a block off or miles away.

If there is not good health, the brain inevitably partakes of its share of disease, and works imperfectly or wildly, according to circumstances; and the sermon or lecture, fabricated under such circumstances, is infallibly deficient in the clearness of its logic, as well as in the wisdom and moderation of its application. "A sound mind in a sound body," is a maxim thousands of years old, and as true as it is ancient.

What then is the cause of this ill-health in the clergy? Who ever heard of a clergyman, except it be the hardy and self-denying Methodist circuit-rider, who could not call every finger to count an ailment. A perfectly healthy clergyman among the educated sects, is a "rare bird." None of the other learned professions can muster so large an army of invalids, in proportion to the whole number, as the clerical, and there must be a cause for it. That cause is, in our opinion, two-fold:

Inattention to their habits of life.

Insufficient ministerial support.

Of the two, the latter is the more controlling, because it wears out the body, soul, and spirit together.

No lawyer, or physician, or artist, ever rose to eminence, who did not consecrate his whole time, bodily, and his whole energies, mentally, to the prosecution of his calling; and yet to be competent to his place, a clergyman must have a far wider range of learning than either of these, because he has to come in collision with all men everywhere—alike with the philosopher and the fool, the countryman and the courtier, the savage and the sage.

The greatest among all lawyers, and the most skillful among all physicians, are those who have not devoted their entire energies to their general professions, but who have concentrated them all on some one particular branch, as surgery and criminal law; but no such facilities for greatness are allowed a clergyman; the range of his investigations is world wide; the field which he has to scan is as boundless as the universe; it embraces eternity, as well as all time.

To become a master workman then, a clergyman must have his whole time consecrated to his calling; and to do this, he must be so well paid, as to relieve him altogether from the slightest anxiety as to an adequate, a generous, a full support for himself, and for all who are dependent on him. That any man of ordinary intelligence should not, at first glance, see and feel the reasonableness of the sentiment, must be attributed to the fact, that *he*, at least, gives so much of his attention to his own particular calling, that he has not time to think much about church matters, as "his business requires *all* his care!"

It may be useful, in this connection, to look at a fact or two. Who are the ablest and longest lived among the educated clergy of all denominations? We reply, without fear of contradiction as to the generality of the rule, *they are the men who have the largest salaries.* Within half a mile of this very "Forty Two, Irving Place, New York," we can count nearly a dozen churches, of various denominations, whose ministers are famed for their piety, their learning, their efficiency; men of a wide-spread reputation. They are all men beyond middle age, men of gray hairs, averaging from three to five thousand a year; and when the session of one of them endeavored to force another thousand per annum on him, he wouldn't have it, saying, "I am comfortable enough with my present salary," and a better man, a harder worker, and more efficient, and withal, a more unpretending, we do not know in all this land.

The true friends of the Church may rest assured, that the most efficient method of eating out all that is nourishing, and rousing, and sparkling, and bright, in their ministers' discourses, is to put him on half allowance, on a meagre salary, tardily paid; if, indeed, it is ever wholly made up.

On the other hand, take any young man of piety, and of an ordinary seminary education, put him anywhere, pay him so well that he may feel himself a man in any crowd, with no consciousness of an unpaid obligation, with no fear of having to borrow; let him feel that his compensation will certainly be paid at the day, and in full, and take our word for it, that it will not be long before you had rather see him ascend the pulpit on the Sabbath day, than any other man; and, in addition to all, you will feel yourself growing every week in the wideness of your views, and in the stability of your doctrines, your only fear being that he will be taken away from you to fill some higher place.

As to the *ecclesiastical* part of the argument, we will not express our sentiments just now, not wishing to jar many of our personal friends. We could very naturally make it "come in" the pages of our Journal, but we will wait awhile, merely saying, that we are not unsound as to any of the cardinal points of orthodoxy; it is a mere matter of practice and policy, as to the education of the children of the Church. To announce our sentiments on this subject, in plain Anglo-Saxon phrase, would cost any orthodox clergyman in New York his pulpit, probably; and were any of our leading religious journals to avow them, it would be an avowal more costly than that of the editor who declared his readiness to receive as a gift, any well-stocked cotton plantation, the price of which pithy assertion being, as reported, a loss of ten thousand subscribers.

To make this article more complete, we will step out of our way to say, that we have no right "*To take the children's bread and to cast it to dogs*"—no right to extract from "the milk," the religious instruction of our children, that which makes it the "milk," that which gives it its distinctive character.

We have no right, as we practically do, to a great extent, to depute the religious instruction of our children to anybody—not even to the minister, exclusively; that is the duty of the father and mother, so that the holiest memories of a departed mother's affection may be so entwined with her religious teachings, that in after-life their separation may be impossible, that one may sustain the other, that both may be cultivated and cherished and fed together; and this is the foundation of the exceedingly comforting assurance to any Christian parent in the hour of dissolution: "*When he is old, he will not depart from it.*"

In proportion as a man believes his own Church the best, all things considered, as he would give his children the best, he is bound to make the characteristic tenets of his religious faith a part and parcel of his child's education, *first of all things*, to begin before the A B C of the primer; to begin as soon as the child can lisp a yes or no, or point its finger to the stars, in recognition of *their* great Creator, and *its* Father. The regulars of the army are the only ones on which a veteran general relies in the breach of death; and so it is in these days of insidious errors and sweeping delusions. You can never feel sure of finding any man in his place, but him whose mother and father prayerfully taught him the A B C of his religion, and ingrained into his young mind the distinctive doctrines of his Church.

The soldier who would as lief fight under one flag as another, is but a degree better than a traitor. The politician who considers one party as good as another, is but one remove from being a weight on either. A physician who believes there is good in all "pathies," and is ready to practice either, according to the wishes of his patient, might just as well close his office; while the religionist, who is so liberal in his opinions, that he considers any other Church as good as his own, is unworthy of the denomination which has the misfortune to claim him as a member. To be efficient anywhere, we must be decided in sentiment as well as in action; confident first, aggressive next. In homelier phrase, "Be sure you are right, then go ahead." But how can a milk-and-water man accomplish anything anywhere? "*Let every man be fully persuaded in his own mind.*" Upon this is built the doctrine of Aggression, and Aggression is the essence of an active Christianity. But a true enthusiasm in the promulgation of one's own religious sentiments, includes within it a large liberality and charity as to the distinctive tenets of others; while, on the other hand, "exclusiveness," "*Nobody is right but me,*" "*All are in the broad way who are not of my Church,*" is a twin-sister to bigotry, the next-door neighbor to a rude barbarism.

